# Major Role for Amphotericin B–Flucytosine Combination in Severe Cryptococcosis

**DOI:** 10.1371/journal.pone.0002870

**Published:** 2008-08-06

**Authors:** Françoise Dromer, Claire Bernede-Bauduin, Didier Guillemot, Olivier Lortholary, for the French Cryptococcosis Study Group

**Affiliations:** 1 Institut Pasteur, Mycologie Moléculaire, Centre National de Référence Mycologie et Antifongiques, CNRS URA3012, Paris, France; 2 INSERM U657, Paris, France; 3 Institut Pasteur, Pharmacoépidémiologie et Maladies Infectieuses, Paris, France; 4 Faculté de Médecine Paris Ile de France Ouest, Université Versailles Saint Quentin, Versailles, France; 5 Université Paris-Descartes, Hôpital Necker-Enfants Malades, Service des Maladies Infectieuses et Tropicales, Centre d'Infectiologie Necker-Pasteur, Paris, France; Massachusetts General Hospital, United States of America

## Abstract

**Background:**

The Infectious Diseases Society of America published in 2000 practical guidelines for the management of cryptococcosis. However, treatment strategies have not been fully validated in the various clinical settings due to exclusion criteria during therapeutic trials. We assessed here the optimal therapeutic strategies for severe cryptococcosis using the observational prospective CryptoA/D study after analyzing routine clinical care of cryptococcosis in university or tertiary care hospitals.

**Methodology/Principal Findings:**

Patients were enrolled if at least one culture grew positive with *Cryptococcus neoformans*. Control of sterilization was warranted 2 weeks (Wk2) and 3 months (Mo3) after antifungal therapy onset. 208 HIV-positive or -negative adult patients were analyzed. Treatment failure (death or mycological failure) at Wk2 and Mo3 was the main outcome measured. Combination of amphotericin B+flucytosine (AMB+5FC) was the best regimen for induction therapy in patients with meningoencephalitis and in all patients with high fungal burden and abnormal neurology. In those patients, treatment failure at Wk2 was 26% in the AMB+5FC group vs. 56% with any other treatments (p<0.001). In patients treated with AMB+5FC, factors independently associated with Wk2 mycological failure were high serum antigen titer (OR [95%CI] = 4.43[1.21–16.23], p = 0.025) and abnormal brain imaging (OR = 3.89[1.23–12.31], p = 0.021) at baseline. Haematological malignancy (OR = 4.02[1.32–12.25], p = 0.015), abnormal neurology at baseline (OR = 2.71[1.10–6.69], p = 0.030) and prescription of 5FC for less than 14 days (OR = 3.30[1.12–9.70], p = 0.030) were independently associated with treatment failure at Mo3.

**Conclusion/Significance:**

Our results support the conclusion that induction therapy with AMB+5FC for at least 14 days should be prescribed rather than any other induction treatments in all patients with high fungal burden at baseline regardless of their HIV serostatus and of the presence of proven meningoencephalitis.

## Introduction

Major information on the best therapeutic strategies for cryptococcal meningoencephalitis derives from therapeutic trials involving HIV-positive [1], [2], [3] or HIV-negative patients [4]. According to the current Infectious Diseases Society of America (IDSA) guidelines, the treatment should depend on anatomic site and host's immunological status. Induction therapy using a combination of amphotericin B (AMB, 0.7–1 mg/kg/d) and flucytosine (5FC, 100 mg/kg/d) for 2 weeks followed by a consolidation phase of 10 weeks by fluconazole (FCZ, 400 mg/d) should be prescribed for central nervous system infection (CNS) in both HIV-positive and -negative patients, based mostly on data extrapolated from trials in HIV-infected patients [5] and retrospective studies on HIV-negative patients [6], [7]. Alternative options to the AMB+5FC combination are advocated and the place of 5FC is still questioned by numerous clinicians especially in HIV-negative patients or in case of mild-to-moderate symptoms, even if AMB+5FC combination therapy is the most fungicidal regimen for CNS infections [8]. Since they are excluded from therapeutic trials, data are missing on the optimal antifungal treatment for non CNS infections and for the most severe cases of meningoencephalitis. Finally, the trials design made mandatory the switch from induction to consolidation treatment on day 15. Thus, culture results for samples collected at that time are not taken into account whereas they influence subsequent outcome [9].

Using data from a prospective observational cohort of HIV-positive and -negative patients treated in France for cryptococcosis with or without CNS involvement, we previously showed that lack of 5FC was an independent factor of mycological failure at Wk2 whatever the HIV status and the clinical presentation at baseline [10]. We also defined criteria of severity at baseline that go beyond the documentation of CNS infection or abnormal neurology. We here analyzed the impact of antifungal treatment strategies prescribed in routine clinical practice on outcome 2 and 12 weeks after the diagnosis of cryptococcosis, based on appraisal of CNS involvement, IDSA guidelines and our previously defined severity criteria [10].

## Methods

### Study design

The CryptoA/D study enrolled HIV-positive or -negative adults experiencing a first episode of culture-proven cryptococcosis and treated in French university hospitals or tertiary care centers [10]. The design of the study has already been detailed [10]. Briefly, for each patient, a workup including culture of blood, cerebrospinal fluid (CSF) and urine was requested at baseline to systematically evaluate fungal burden and dissemination. Controls of sterilisation for all initially infected body sites (i.e. those with positive culture) were systematically done two weeks (Wk2) and three months (Mo3) after the onset of antifungal therapy. Other investigations and all therapeutic decisions (choice of drugs, duration and dosage) were left to the clinician in charge. All data were recorded until Mo3 through a standardized questionnaire by the local investigators. Missing information was systematically checked with both the clinician and the microbiologist. The study was approved by the local ethical committee and notified to the French Ministry of Health (registration # DGS970089). Written informed consent was obtained for all patients.

### Definitions

A case was defined by isolation of *C. neoformans* from at least one body site. Cases were classified as meningoencephalitis (*C. neoformans*-positive CSF culture, positive direct examination, and/or CSF antigen testing) or as extrameningeal cryptococcosis. A threshold of ≥1∶512 for serum or CSF antigen titer was selected since it is linked to prognosis [10] and subsequent relapse [11]. Abnormal neurology was defined by presence of seizures, abnormal mental status and/or neurological defect. Results of brain imaging were reported after evaluation by the local radiologist. Our criteria of severity included abnormal neurology, meningoencephalitis, fungemia, dissemination, high antigen titer [10]. Induction therapy defined the regimen prescribed between baseline and the Wk2 evaluation. Consolidation therapy followed the Wk2 workup. Only prescriptions lasting at least 5 days were taken into account. Optimal induction treatment was defined for AMB and 5FC in terms of dosage (0.7–1 mg/kg/d for AMB and 100±15 mg/kg/d for 5FC) or duration (14±2 days) according to the 2000 IDSA guidelines [5]. Intralipids did not modify the optimal dosage of AMB, whereas 5mg/kg/d of amphotericin B lipid complex (Abelcet®) and 3 mg/kg/d of liposomal amphotericin B (Ambisome®) were required to be considered optimal. Cumulative doses were calculated as: [duration in days×daily dosage in mg/kg×weight in kg].

Mycological outcome was only evaluated in patients for whom at least one body site was sampled at the time of workup. Mycological failure meant that at least one of the cultured samples contained viable yeasts. Mycological failure or death up to 4 days after Wk2 workup was recorded as treatment failure at Wk2. Mycological failure or later death was recorded as treatment failure at Mo3.

### Statistical analysis

We first described and compared the major characteristics of the patients and the Wk2 outcome according to the induction therapy strategies. We then assessed how the current IDSA guidelines [5] were followed and the factors influencing treatment's choice. Finally, we investigated the determinants of Wk2 and Mo3 outcomes.

Clinical presentation and outcome were compared according to therapeutic regimens with the χ2 test for qualitative parameters (or the Fisher's exact test when necessary) or the Kruskal-Wallis's or the Mann-Whitney's test for quantitative parameters. Multinomial logistic regression [12] was used to analyze the influence of socio-demographic and clinical characteristics on induction therapy (AMB or FCZ vs. AMB+5FC) in the assessable population as a whole and in the subpopulation of HIV-positive patients with meningoencephalitis. Logistic regression models were performed to investigate the factors associated with mycological failure at the Wk2 workup and those associated with treatment failure at the Mo3 workup [13]. All regression final models were obtained using the backward procedure. First, all covariates with a p-value under 0.25 in univariate analysis and 2 order-interactions with a p-value under 0.05 were simultaneously entered into the regression model. Next, the set of covariates with the largest p-value was iteratively removed from the model until all of the covariates (or blocks of covariates) remaining in the reduced model had a p-value under 0.05. A p-value of <0.05 was considered statistically significant. Statistical analyses were performed with Stata software©, Version 9.0.

## Results

### Population

**Figure 1:**
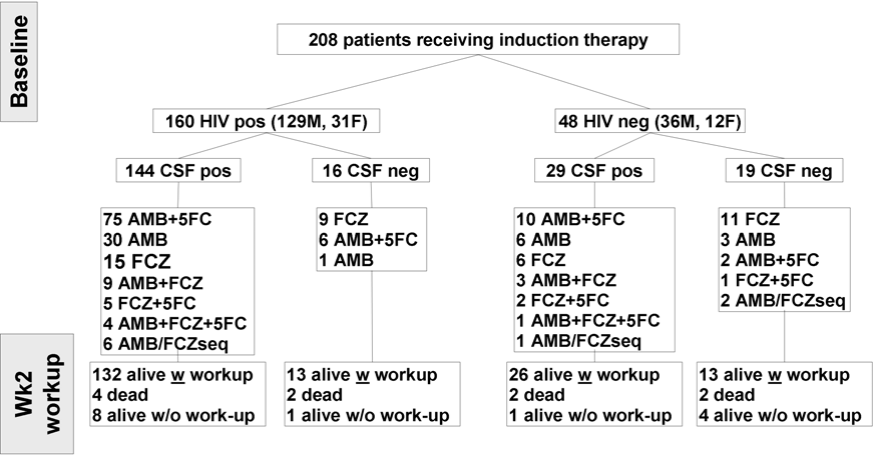
Flow chart showing the actual number of patients with cryptococcosis included in the description of clinical presentation, determinant of induction treatment choices and analysis of week 2 (Wk2) outcome. HIV-positive and -negative patients with cryptococcal meningoencephalitis (CSF pos) or with no proven central nervous system involvement (CSF neg) were enrolled in the prospective multicenter CryptoA/D observational study. The Wk2 workup requested control of sterilisation of the body sites infected with *C. neoformans* at baseline. Treatments prescribed for more than 5 days included amphotericin B (AMB), flucytosine (5FC) and/or fluconazole (FCZ). Overall, of the 208 patients eligible for analysis at baseline, at the time of the Wk2 workup, 184 were alive with mycological outcome assessed, 14 were alive without mycological evaluation and 10 had died.

**Figure 2:**
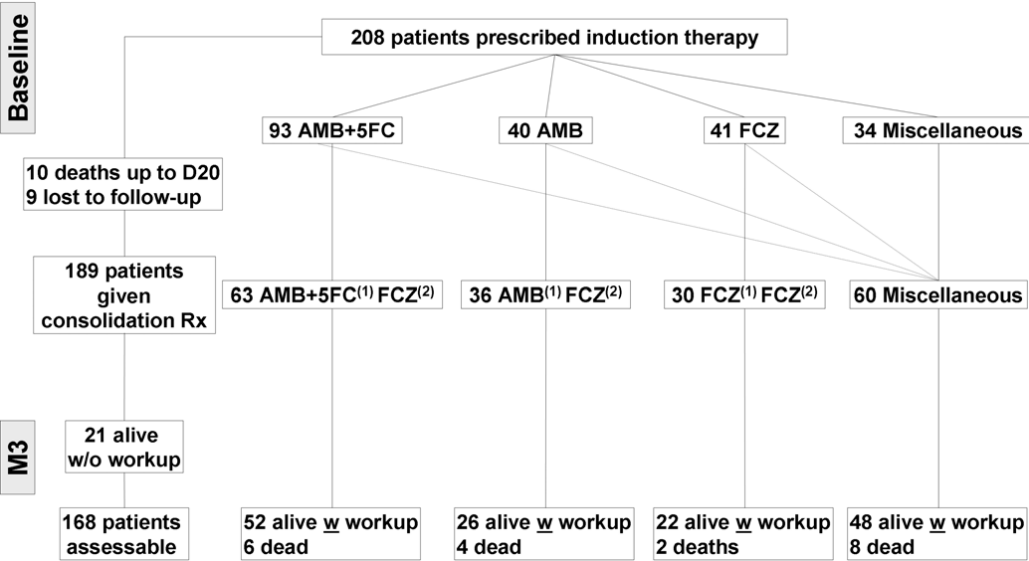
Flow chart showing the actual number of patients with cryptococcosis included in the description and analysis of the consolidation phase and outcome 3 months (Mo3) after onset of antifungal therapy. Of the 208 patients enrolled initially, 10 died during the induction phase and 9 were lost to follow-up leaving 189 patients for the description of disease and outcome. At the Mo3 workup, 168 patients were assessable including 148 patients alive with mycological assessment and 20 who had died. The remaining 21 patients alive were not included in the final analysis since they had no mycological evaluation at the Mo3 workup.

Induction therapy was assessable for 208 patients including a majority of HIV-positive male patients with meningoencephalitis (Table 1, Figure 1). At the time of the Wk2 workup, 10 patients had died, and 184/198 patients alive were assessable for mycological outcome. At the time of the Mo3 workup, 9 patients were lost to follow-up, 20 had died, and 148/169 patients alive were available for mycological evaluation (Figure 2).

**Table 1:** Description of the population and comparison of induction therapy regimen according to selected parameters of clinical presentation and outcome at Wk2 in patients with cryptococcosis (n = 208).

	All patients	Patients receiving one of the following regimen, no./no. evaluated (%)							P^a^
		AMB+5FC	AMB	FCZ	Other strategies				
					AMB+FCZ	AMB/FCZ seq	FCZ+5FC	AMB+FCZ+5FC	
	n = 208	n = 93	n = 40	n = 41	n = 12	n = 9	n = 8	n = 5	
**Baseline parameters**									
Mean age in years±SD	43±13	39±11	42±13	47±15	50±12	50±14	45±8	35±9	<0.001
Male gender	165/208 (79)	70/93 (75)	35/40 (88)	33/41 (80)	11/12 (92)	6/9 (67)	6/8 (75)	4/5 (80)	0.585
HIV infection	160/208 (77)	81/93 (87)	31/40 (78)	24/41 (59)	9/12 (75)	6/9 (67)	5/8 (63)	4/5 (80)	0.022
Haematological malignancy	18/208 (9)	3/93 (3)	4/40 (10)	6/41 (15)	3/12 (25)	2/9 (22)	0/8 (0)	0/5 (0)	0.046
Born in Africa	59/207 (28.5)	31/93 (33)	12/39 (31)	10/41 (24)	3/12 (25)	3/9 (33)	0/8 (0)	0/5 (0)	0.334
Living in Paris area	117/208 (56)	61/93 (66)	22/40 (55)	20/41 (49)	5/12 (42)	4/9 (44)	2/8 (25)	3/5 (60)	0.173
Current or past smoker	115/198 (58)	51/91 (56)	19/35 (54)	23/40 (57)	7/11 (64)	4/8 (50)	6/8 (75)	5/5 (100)	0.51
Tertiary care hospital	61/208 (29)	23/93 (25)	10/40 (25)	12/41 (29)	7/12 (58)	3/9 (33)	2/8 (25)	1/5 (20)	0.051
Abnormal neurology	81/208 (39)	40/93 (43)	16/40 (40)	9/41 (22)	6/12 (50)	3/9 (33)	4/8 (50)	3/5 (60)	0.259
Natremia<134 mEq/L	110/204 (54)	51/92 (55)	23/39 (59)	17/39 (44)	6/12 (50)	6/9 (67)	6/8 (75)	1/5 (20)	0.362
Meningoencephalitis	173/198 (87)	85/91 (93)	36/39 (92)	21/35 (60)	12/12 (100)	7/8 (88)	7/8 (88)	5/5 (100)	<0.001
Abnormal brain imaging,	47/170 (28)	31/83 (37)	7/34 (21)	4/23 (17)	2/11 (18)	1/8 (13)	1/7 (14)	1/4 (25)	0.237
Fungemia	78/203 (38)	45/93 (48)	16/38 (42)	8/39 (21)	3 /11 (27)	3/9 (33)	1/8 (13)	2/5 (40)	0.054
High (≥1∶512) serum antigen titer	85/194 (44)	50/89 (56)	17/38 (45)	6/40 (15)	4/8 (50)	3/7 (43)	2/7 (29)	3/5 (60)	0.002
High (≥1∶512) CSF antigen titer	72/165 (44)	49/83 (59)	11/33 (33)	4/26 (15)	4/8 (50)	1/5 (20)	2/6 (33)	1/4 (25)	0.003
Positive India ink staining	140/172 (81)	75/85 (88)	28/36 (78)	12/21 (57)	10/11 (91)	5/7 (71)	6/7 (86)	4/5 (80)	0.056
**Induction treatments**									
Optimal duration and dosage for AMB	94/159 (59)	63/93 (68)	22/40 (55)	-	6/12 (50)	0/9 (0)	-	3/5 (60)	
Optimal duration and dosage for 5FC	35/106 (33)	32/93 (34)	-	-	-	-	1/8 (13)	2/5 (40)	
Mean cumulative dose of AMB±SD (mg)	871±720	831±632	937±685	-	1399±1363	474±236	-	535±269	0.006
Mean cumulative dose of FCZ±SD (g)	7±3	-	-	7.5±2.8	6.1±3.5	4.5±3.4	7.6±2.6	3.8±2.4	0.003
Mean cumulative dose of 5FC±SD (g)	87±40	90±41	-	-	-	-	61±40	71±23	0.092
AMB in intralipids/Ambisome/ABLC, n/n/n	31/10/2007	15/6/1	10/2/5	-	2/2/1	2/0/0	-	2/0/0	
**Parameters at the Wk2 workup**									
Wk2 work-up performed	184	86	34	35	10	9	7	3	
CSF positive India ink staining at Wk2	96/147 (65)	57/74 (77)	17/31 (55)	8/19 (42)	7/9 (78)	1/5 (20)	4/6 (67)	2/3 (67)	0.016
Mycological failure in patients alive at the Wk2 workup:									
all patients	66/184 (36)	20/86 (23)	20/34 (59)	14/35 (40)	6/10 (60)	2/9 (22)	2/7 (29)	2/3 (67)	0.005
patients with meningoencephalitis	64/158 (40.5)	20/80 (25)	20/32 (63)	12/20 (60)	6/10 (60)	2/7 (29)	2/6 (33)	2/3 (67)	0.002
patients with meningoencephalitis and abnormal neurology	32/72 (44)	9/39 (23)	10/13 (77)	6/8 (75)	4/5 (80)	1/2 (50)	1/3 (33)	1/2 (50)	0.005
Treatment failure at Wk2^b^	76/194	23/89 (26)	23/37 (62)	16/37 (43)	8/12 (67)	2/9 (22)	2/7 (29)	2/3 (67)	0.002

Abbreviations: AMB amphotericin B, FCZ fluconazole, 5FC flucytosine, AMB/FCZseq: sequential administration of AMB then FCZ.

a global comparison of the 7 treatment groups.

b presence of viable yeasts at the Wk2 work-up or death up to day 20.

### Induction therapy and its influence on outcome of patients with cryptococcosis

Induction therapies included AMB (159/208, 76%) mostly associated with 5FC (98/159, 62%) or FCZ (75/208, 36%) mostly alone (41/75, 55%) (Table 1). AMB was sometimes administered in intralipids (n = 31), liposomal amphotericin B (n = 10) or amphotericin B lipid complex (n = 7). French clinicians followed the IDSA guidelines (AMB+5FC) for 85/173 (49%) of the patients with meningoencephalitis. Only 87 (46.5%) of the 187 patients fulfilling our severity criteria [10] received the AMB+5FC combination. Prescriptions were not different in tertiary care centers or university hospitals (data not shown).

Despite more severe infections based on our criteria, mycological failure at Wk2 was significantly less frequent among patients treated with AMB+5FC than any other regimen (20/86 (23%) vs. 47/100 (47%), p<0.001) (Table 1). This was still true with the same yield of sterilisation for patients with meningoencephalitis and even those with abnormal neurology at baseline, whereas in the case of abnormal neurology the percentage of mycological failure increased from 42% to 75% in patients treated with FCZ alone, and from 59% to 77% in those treated with AMB alone. The highest rate of mycological failure was observed for AMB alone. Addition of AMB to FCZ did not better than either drug alone. The small number of patients treated with FCZ+5FC (n = 8) or AMB+FCZ+5FC (n = 5) prevents definitive conclusion but none of these treatments did better than AMB+5FC, especially in the most severe cases. There was no statistical differences for patients treated by AMB+5FC with AMB in lipid formulations (n = 22) or AMB deoxycholate (n = 71) in terms of disease severity or outcome (data not shown). When we analyzed the proportion of treatment failures, AMB+5FC did better than any other treatment regimen in patients with meningoencephalitis and abnormal neurology (10/40 (25%) vs. 26/36 (72%), p<0.001). With extended criteria of severity, AMB+5FC still did better than any other regimen (22/84 (26%) vs. 52/93 (56%), respectively, p<0.001). In the multivariate analysis involving all patients (n = 171), independent parameters of treatment failures at Wk2 were lack of induction therapy with AMB+5FC vs. any other induction therapies (OR [95% CI] = 5.16 [2.44–10.91], p<0.0001), presence of meningoencephalitis (OR = 4.45 [1.34–14.79], p = 0.015) and high serum antigen titer (OR = 2.99 [1.43–6.24], p = 0.003) at baseline. In the multivariate analysis involving patients with meningoencephalitis (n = 123), independent parameters of treatment failures at Wk2 were lack of induction therapy with AMB+5FC vs. any other induction therapies (OR [95% CI] = 51.25 [9.67–271.52], p<0.0001), abnormal brain imaging (OR = 4.18 [1.33–123.17], p = 0.014), presence of fungemia (OR = 3.07 [1.13–8.31], p = 0.027) and high CSF antigen titer [17.80 [3.79–83.54], p<0.0001) at baseline. For both models (all patients or patients with meningoencephalitis), interactions between covariates were checked. None was significant.

### Determinants of induction therapy's choice

The majority of patients (n = 174) were treated with AMB or FCZ alone, or AMB+5FC. Patients treated with AMB did not significantly differ from those treated with the combination therapy except for the lower percentage of patients with high CSF antigen titer in the AMB-treated group (Table 2). By contrast, compared to patients treated with the combination, the proportion of HIV-infection, abnormal neurology, meningoencephalitis, fungemia, high serum or CSF antigen titers was significantly lower in patients treated with FCZ whereas the proportion of older patients was higher. In the multinomial logistic regression comparing the 3 treatment groups with the AMB+5FC combination as the reference, the presence of high CSF antigen titers, and an age under 40 years were the only factors associated with the prescription of the combination vs. FCZ alone and only the presence of high CSF antigen titers was determinant for the prescription of the combination vs. AMB alone.

**Table 2:** Determinants of prescription of amphotericin B (AMB, n = 40), fluconazole (FCZ, n = 41) or the combination of amphotericin B and flucytosine (AMB+5FC, n = 93) for induction therapy of culture-proven cryptococcosis in HIV-positive and -negative patients (n = 174).

	Univariate OR [CI95%]	P	Adjusted OR [CI95%]	P
	AMB vs. AMB+5FC		AMB vs. AMB+5FC	
	FCZ vs. AMB+5FC		FCZ vs. AMB+5FC	
HIV infection	0.51 [0.20–1.33]	0.169		
	0.21 [0.09–0.50]	<0.001		
>40 years-old	1.63 [0.76–3.51]	0.210	1.30 [0.54–3.12]	0.552
	4.26 [1.95–9.29]	<0.001	4.62 [1.70–12.58]	0.003
Male gender	2.3 [0.81–6.56]	0.120		
	1.36 [0.55–3.35]	0.510		
Born in Africa	0.89 [0.40–1.99]	0.774		
	0.65 [0.28–1.48]	0.302		
Living in Paris area	0.64 [0.30–1.37]	0.249		
	0.50 [0.24–1.05]	0.069		
Current or past smoker	0.93 [0.43–2.04]	0.859		
	1.06 [0.50–2.25]	0.877		
Tertiary care hospital	1.01 [0.43–2.39]	0.974		
	1.26 [0.55–2.86]	0.582		
Abnormal neurology	0.88 [0.42–1.88]	0.747		
	0.37 [0.16–0.87]	0.022		
Natremia <134 mEq/L	1.16 [0.54–2.47]	0.709		
	0.62 [0.29–1.32]	0.216		
Meningoencephalitis	0.85 [0.20–3.57]	0.821		
	0.11 [0.04–0.31]	<0.001		
Fungemia	0.78 [0.36–1.66]	0.513		
	0.28 [0.11–0.66]	0.004		
Abnormal brain imaging	0.43 [0.17–1.12]	0.083		
	0.35 [0.11–1.13]	0.080		
High (≥1∶512) CSF antigen titer	0.35 [0.15–0.81]	0.014	0.34 [0.15–0.80]	0.013
	0.13 [0.04–0.40]	<0.001	0.11 [0.03–0.36]	<0.001
High (≥1∶512) serum antigen titer	0.63 [0.29–1.36]	0.238		
	0.14 [0.05–0.36]	<0.001		

In HIV-infected patients with meningoencephalitis who received as induction therapy either AMB or FCZ alone or the combination AMB+5FC (n = 120), factors which significantly discriminated between combination and FCZ alone in the univariate analysis were the presence of fungemia, high serum or CSF antigen titer, and hospitalization in university hospitals with a trend towards modification of prescription in older patients, while only high CSF antigen titer was identified as a factor associated with the initiation of combination therapy vs. AMB alone (Table S1). In the multinomial logistic regression, the presence of high CSF antigen titer and an age under 40 were independently associated with combination therapy vs. FCZ alone while only high CSF antigen titer was determinant for the prescription of AMB+5FC compared to AMB alone.

### Determinants of early outcome (Wk2) after induction therapy with the AMB+5FC combination therapy

Among the 86 patients given AMB+5FC and assessable at Wk2, the proportion of men, and high serum or CSF antigen titer was significantly higher than with other treatments. There was also a trend towards more disseminated infections and infection by serotype A among those who experienced mycological failure at Wk2 than among those who were cured. An optimal duration and dosage of each drug in the combination had no impact. Factors independently associated with mycological failure at Wk2 were high serum antigen titer (OR [95%CI] = 4.43 [1.21–16.23], p = 0.025) and abnormal brain imaging (OR [95%CI] = 3.89 [1.23–12.31], p = 0.021) at baseline (Table 3).

**Table 3:** Parameters associated with mycological failure (persistence of at least one body site infected) in patients alive at the Wk2 workup and treated with combination therapy of AMB+ 5FC (n = 86): univariate and multivariate analyses.

Parameter	No./No. evaluated (%) of patients		Univariate OR	P	Adjusted OR	P
	Mycological cure (n = 66)	Mycological failure (n = 20)	[CI95%]		[CI95%]	
Less than 14 days of combination	28/66 (42)	9/20 (45)	1.11 [0.40–3.06]	0.8394		
Non optimal dosage of both drugs	31/66 (47)	11/20 (55)	1.38 [0.50–3.80]	0.5315		
Male gender	48/66 (73)	19/20 (95)	7.13 [0.83–61.22]	0.0365		
Fungemia	29/66 (44)	13/20 (65)	2.37 [0.82–6.86]	0.1008		
High (≥1∶512) serum antigen titer	32/64 (50)	15/19 (79)	3.75 [1.07–13.12]	0.0263	4.43 [1.21–16.23]	0.025
High (≥1∶512) CSF antigen titer	30/61 (49)	17/17 (100)	-	<0.001		
Abnormal neurology	30/66 (45)	9/20 (45)	0.98 [0.36–2.70]	0.9716		
Serotype A	50/61 (82)	19/19 (100)	-	0.058		
Abnormal brain imaging	19/59 (32)	11/20 (55)	2.57 [0.89–7.47]	0.0713	3.89 [1.23–12.31]	0.021

### Strategies of consolidation therapy and their influence on Mo3 outcome

Of 189 patients surviving the Wk2 workup by more than 4 days, 168 were eligible for analysis of Mo3 outcome (Figure 2). The induction treatment was sometimes protracted in the consolidation phase. Four groups were considered: FCZ all along (FCZ^(1)^FCZ^(2)^), AMB followed by FCZ (AMB^(1)^FCZ^(2)^), AMB+5FC followed by FCZ (AMB+5FC^(1)^FCZ^(2)^) and “miscellaneous” corresponding to other induction therapies and/or multiple changes preventing further description.

Considering the first 3 treatment groups, patients in the AMB+5FC^(1)^FCZ^(2)^ group had significantly the more severe infections, and those in the FCZ^(1)^FCZ^(2)^ group the less severe ones (Table S2). Mycological failure was 2.5-fold less frequent in the AMB+5FC^(1)^FCZ^(2)^ group (20%) than in the AMB^(1)^FCZ^(2)^ group (52%) despite no significant difference in the cumulative dose and duration of AMB. Of note, direct examination of the Wk2 CSF samples was still positive in almost 3/4 and 1/2 of the cases in AMB+5FC^(1)^FCZ^(2)^ and AMB^(1)^FCZ^(2)^ treated patients, respectively. There was a trend toward less treatment failures recorded at Mo3 in the AMB+5FC^(1)^FCZ^(2)^ group than in the others.

In the univariate analysis, the proportion of abnormal neurology at baseline, haematological malignancies and 5FC prescription lasting less than 14 days was significantly higher in patients recorded as treatment failures at Mo3 than in the others (Table 4). There was a trend towards a higher proportion of mycological failure at the Wk2 workup in those who were subsequently recorded Mo3 failures than in those who were not. Mean duration and cumulative dose of FCZ were significantly lower in case of treatment failure at Mo3 compared to treatment success, as expected since FCZ duration was shorter in patients dying between Wk2 and Mo3. In the multivariate analysis haematological malignancy (OR [95%CI] = 4.02 [1.32–12.25], p = 0.015), abnormal neurology at baseline (OR [95%CI] = 2.71 [1.10–6.69], p = 0.030) and prescription of 5FC for less than 14 days (OR [95%CI] = 3.30 [1.12–9.70], p = 0.030) were independently associated with treatment failure at Mo3.

**Table 4:** Parameters associated with treatment failure at the Mo3 workup in 168 HIV-positive and -negative patients with cryptococcosis: univariate and multivariate analyses.

Parameter	No./No. evaluated (%) of patients		Univariate OR [CI95%]	P	Adjusted OR [CI95%]	P
	Treatment cure (N = 142)	Treatment failure (N = 26)				
**Baseline parameters**						
>40 years-old	61/142 (43)	12/26 (46)	1.14 [0.49–2.64]	0.763		
Male gender	113/142 (80)	22/26 (85)	1.41 [0.45–4.44]	0.553		
HIV infection	112/142 (79)	19/26 (73)	0.73 [0.28–1.90]	0.513		
Haematological malignancy	10/142 (7)	7/26 (27)	4.86 [1.60–14.82]	0.002	4.02 [1.32–12.25]	0.015
Abnormal neurology	52/142 (37)	15/26 (58)	2.36 [1.00–5.59]	0.044	2.71 [1.10–6.69]	0.030
Natremia (<134 mEq/L)	75/140 (54)	10/26 (38)	0.54 [0.23–1.29]	0.158		
Meningoencephalitis	118/136 (87)	23/25 (92)	1.75 [0.38–8.14]	0.467		
Abnormal brain imaging	29/118 (25)	8/22 (36)	1.75 [0.66–4.64]	0.251		
Fungemia	56/140 (40)	9/25 (36)	0.84 [0.35–2.05]	0.707		
High (≥1∶512) CSF antigen titer	55/119 (46)	8/20 (40)	0.78 [0.29–2.04]	0.607		
High (≥1∶512) serum antigen titer	59/133 (44)	12/25 (48)	1.16 [0.49–2.73]	0.738		
Serotype A isolate	103/139 (74)	18/26 (69)	0.79 [0.31–1.97]	0.607		
**Wk2 workup**						
Positive India ink staining of the CSF	76/109 (70)	12/19 (63)	0.74 [0.27–2.07]	0.570		
Mycological failure	44/135 (33)	12/23 (52)	2.26 [0.91–5.58]	0.071		
**Treatment strategies for induction and consolidation phases** a						
Treatment composed of AMB+5FC^(1)^ FCZ^(2)^	52/142 (37)	6/26 (23)	-	-		
Treatment composed of FCZ^(1)^ FCZ^(2)^	20/142 (14)	4/26 (15)	1.73 [0.44–6.80]	0.430		
Treatment composed of AMB^(1)^ FCZ^(2)^	25/142 (18)	5/26 (19)	1.73 [0.48–6.23]	0.399		
Other treatments	45/142 (32)	11/26 (42)	2.12 [0.73–6.19]	0.170		
<14 days AMB+5FC	89/142 (63)	21/26 (81)	2.50 [0.88–7.12]	0.075		
<14 days AMB	51/142 (36)	7/26 (27)	0.66 [0.26–1.68]	0.377		
<14 days 5FC	80/142 (56)	21/26 (81)	3.26 [1.14–9.31]	0.020	3.30 [1.12–9.70]	0.030
Mean AMB duration in days±SD	24±16 (n = 115)	23±13 (n = 21)	-	0.88		
Mean AMB cumulative dose in mg±SD^b^	1758±2412 (n = 115)	2139±2563 (n = 20)	-	0.80		
Mean 5FC duration in days±SD	23±20 (n = 88)	14±4 (n = 13)	-	0.06		
Mean 5FC cumulative dose in g±SD	148±135 (n = 88)	103±55 (n = 12)	-	0.35		
Mean FCZ duration in days±SD	68±22 (n = 141)	40±24 (n = 24)	-	<0.001		
Mean FCZ cumulative dose in g±SD	32±15 (n = 141)	20±13 (n = 24)	-	<0.001		

Abbreviations: CSF cerebrospinal fluid, FCZ: fluconazole, AMB amphotericin B, 5FC flucytosine

a sequence of prescription indicated by the number in parentheses

b Cumulative dose = duration in days ^*^ daily dosage in mg/kg ^*^ weight in kg.

## Discussion

Cryptococcal meningoencephalitis is still a major health problem in developed countries with the burden of the AIDS epidemics reaching a prevalence of up to 18% in the most severely immunocompromised patients [14]. Almost 20% of the patients die within the first 3 months [10], [15], [16]. That figure has not been changed by the introduction of HAART even if long term survival of HIV-infected patients has increased drastically [11], [17]. Mortality rate is even higher in HIV-negative patients especially for patients with haematological malignancy [7], [10], [18] and in developing countries [15], [19]. Improving the prognosis of cryptococcosis relies upon three factors, and first, the control of the underlying immunodeficiency [20], [21] as shown by the beneficial effect of HAART (with the remaining question of the best timing for its introduction [22]), and preliminary results obtained with administration of gamma interferon or monoclonal antibodies [23], [24]. The other two parameters are a better appraisal of disease severity at baseline and optimization of antifungal treatment strategies which we aim at deciphering here. Results obtained from the analysis of an observational cohort are not biased by a series of entry criteria in therapeutic protocols and show the actual outcomes of cryptococcosis in the general medical community [20]. One should keep in mind however that the study design cannot provide equal figures in each treatment group with the risk of influencing significance. Thus, the most useful and relevant data obtained through observational cohorts are those confirming results established during randomised therapeutic trials. Furthermore, they can guide the redaction of future practical guidelines by showing how clinicians interpret the current recommendations.

Evaluation of cryptococcosis severity represents a major factor for the management of the patients. Many parameters are predictive of treatment failure including underlying malignancy, cranial hypertension, dissemination, and high antigen titer [3], [7], [9], [10], [18], [25]. High serum or CSF antigen titers at baseline have been associated with early mycological failure regardless of the HIV serostatus [10], in HIV-infected patients with meningoencephalitis [9], [26] and in the subgroup of patients treated with AMB+5FC as induction therapy, while high serum antigen titer over time is an independent parameter predictive of cryptococcosis relapse in HIV-infected patients [11].Abnormal brain imaging at baseline previously associated with an increased probability of death [10] was here associated with treatment failure at the Wk2 workup in patients with meningoencephalitis including the subgroup of patients given AMB+5FC, yet considered the optimal induction treatment. Of note, the presence of cryptococcosis-related lesions has been significantly associated with high serum or CSF antigen titer independently of abnormal neurology [27]. Altogether, these data suggest that investigating brain lesions may be critical for optimal management of cryptococcal meningoencephalitis.

Initial antifungal treatment strategy is a critical determinant of outcome. Major studies supporting the prescription of 5FC in combination with AMB for the induction treatment of cryptococcal meningoencephalitis in HIV-positive [2] or -negative [4] patients have been published. Addition of 5FC during the induction phase was an independent factor of sterilisation at Wk2 [2]. AMB+5FC was the most fungicidal treatment for cryptococcal meningitis in Thai patients not receiving HAART [8]. Furthermore, the factor best associated with relapse was the patient having not received flucytosine during the induction phase [28]. One should however keep in mind that therapeutic trials exclude patients with severe abnormal neurology. Furthermore, in routine clinical practice, prescription of 5FC is often restricted to the most severe patients and to patients without haematological disorders(despite limited side effects outside the setting of renal failure ever since the recommended daily dosage has been dropped to 100 mg/kg/d). Finally, treatment is often stopped when the isolate is tested resistant to 5FC, while the combination can still be synergistic in vitro and in vivo [29], [30]. Thus, convincing data showing that AMB+5FC is the optimal treatment in the “real life” is probably warranted. We analyzed what determined the prescription of AMB +5FC versus that of AMB or FCZ alone for induction treatment. In the multinomial logistic regression, the parameter independently associated with prescription of AMB+5FC rather than AMB was a high CSF antigen titer regardless of the HIV serostatus. Those independently associated with the prescription of AMB+5FC rather than FCZ were a high CSF antigen titer and an age under 40 years suggesting that the “older” patients were not given the current best therapeutic option.

We indeed showed that outcome at Wk2 was statistically better with AMB+5FC compared to any other regimen including AMB alone despite more severe infections in the former group. Furthermore, the proportion of Wk2 mycological failure did not increase in the most severe cases treated with AMB+5FC in contrast to what occurred in all other treatment groups except FCZ+5FC. The drastic difference in outcome between the two groups is probably due to the fact that more severe cases than those usually enrolled in the therapeutic trials were considered here with 40% of abnormal neurology compared to less than 20% in previous studies [2], [8]. Furthermore, fungal burden was also increased based on a higher proportion of patients with high antigen titers and positive India ink staining in the AMB+5FC vs. the AMB group. Given the better fungicidal efficacy of AMB+5FC this is likely to have increased the differences. We analyzed how taking into account severity criteria influenced outcomes. Compared to patients treated with other regimens, the percentage of treatment failure was significantly lower in patients with meningoencephalitis treated with the AMB+5FC combination as recommended by IDSA [5]. With criteria of severity extended beyond CNS involvement [10], AMB+5FC still did better than any other regimen. Altogether these data suggest that AMB+5FC is the optimal treatment for severe cryptococcosis.

In the setting of a therapeutical trial on cryptococcal meningoencephalitis in HIV-positive patients, AMB compared favourably with FCZ, the latter being associated with more frequent early deaths and delayed CSF sterilization [1]. Disappointing results were obtained with AMB alone in Thailand [19]. And our data show that, AMB alone did not better than FCZ alone in patients with meningoencephalitis in terms of mycological sterilization at Wk2, both being by far less efficient than AMB+5FC. It is unlikely that this poor efficacy of AMB alone was related to AMB resistance, since very few resistant isolates have been described. Furthermore, there is no correlation between in vitro and in vivo results [31] except with a recently modified broth macrodilution method [32]. Whether this technique can be used routinely and applied to other antifungal drugs remains to be determined. The use of lipid formulations of AMB is another issue. Here, there was no difference in terms of outcome when comparing patients treated with AMB+5FC as AMB deoxycholate or administered in lipid formulations. Use of intralipids was dropped at the end of the 90s because of no substantial beneficial effect over the use of AMB deoxycholate [33]. Data on amphotericin B lipid complex [34] and liposomal AMB [35] are limited. Whether any lipid AMB formulation should be used rather than AMB deoxycholate in the AMB+5FC combination is a real question, often answered empirically to prevent side effects when cost is not a major issue.

When considering the other strategies, our results demonstrate that FCZ alone was associated with 42% mycological failure at Wk2 despite the fact that 40% of the patients in that group did not have meningoencephalitis and that only 15% had high antigen titers. Keeping in mind the small number of patients in each of the FCZ+5FC, AMB+FCZ or AMB+FCZ+5FC groups, none of the combination did better than AMB+5FC. Of note, Bicanic and colleagues recently underlined in South Africa the high relapse rates after treatment with FCZ alone at conventional dosages [36]. Increasing the doses of FCZ improved efficacy of the AMB+FCZ combination in one still unpublished report [37]. In our study, the second best regimen for which mycological failure did not increase with disease severity was FCZ+5FC. Keeping in mind that FCZ is less active in case of high fungal burden [38], one may wonder if increasing FCZ doses could improve efficacy of the FCZ+5FC combination as reported in the experimental model [39]. This alternative regimen should be studied in poor resources areas where AMB use remains difficult. Of note, oral and intravenous formulations of 5FC do not differ in a major way in terms of pharmacokinetics and efficacy [40]. It will require however that 5FC was made commercially available in these countries [21].

Another issue in the optimization of treatment strategies for cryptococcosis is the potential utility of protracting induction regimen. Indeed, current recommendations are based on results obtained in a therapeutic trial where switch from AMB±5FC to azoles was mandatory at Wk2 whatever the culture results at that time [2]. The association between mycological failure at Wk2 and treatment failure at Mo3 demonstrated by Robinson et al.[9] and shown here in an observational cohort as a trend suggest that continuation of the optimal therapy until sterilisation has been demonstrated may be the best option. In addition to recently showing that the lack of 5FC was deleterious for mycological outcome at Wk2 [10], we showed here that less than 14 days of 5FC was independently associated with treatment failure at Mo3. This suggests not only that AMB+5FC should be the first choice for induction treatment in the most severe cases, but that 5FC could be added secondarily if omitted at first and should prescribed for at least 14 days.

Thus, this observational study with its limitation compared to a randomized therapeutic trial but the assets of the “real life” confirms that the AMB+5FC combination is the optimal antifungal strategy for the induction treatment of severe cryptococcosis [5]. In addition, our data strongly suggest that recommending the AMB+5FC combination therapy for at least 14 days for patients with cryptococcosis fulfilling extended criteria of severity [10] decreased the rate of mycological failure at Wk2 and could potentially improve outcome at Mo3.

## Supporting Information

Table S1 (0.02 MB XLS)

Table S2 (0.02 MB XLS)

Appendix S1 (0.03 MB DOC)
