# Supplementary material for: Major Role for Amphotericin B–Flucytosine Combination in Severe Cryptococcosis
Source: PLoS One. 2008 Aug 6;3(8):e2870. doi: 10.1371/journal.pone.0002870 (PMC2483933; doi:10.1371/journal.pone.0002870)
Supplement: Appendix S1 — (0.03 MB DOC) [file pone.0002870.s003.doc]

**APPENDIX S1**

The French Cryptococcosis Study Group is composed of the following individuals who actively participated in the data collection (by alphabetical order of the cities): J. Achard, D. Chabasse (Angers); S. Bland, J.P. Bru (Annecy); M. Pulik, F. Leturdu (Argenteuil); X. Lepeu, H. Lefrand (Avignon); M. Ferrand, M. Larrouy (Bayonne); M. Bentata, C. Bouges-Michel, J. Camuset, L. Guillevin, B. Jarrousse, O. Lortholary, M. Robineau, J.J. Rousset (Bobigny); B. Couprie, M. Dupon, H. Dutronc, J.Y. Lacut, J.L. Pellegrin, J.M. Ragnaud, J.F. Viallard, F.X. Weil (Bordeaux); M.E. Bougnoux, X. Montreal, S. Morelon, E. Rouveix, (Boulogne); P. Granier, H. de Montclos (Bourg-en-Bresse); A. Desveaux, M. Gavignet, A.S. Labussiere, M. Mornet (Bourges); L. De Saint-Martin, E. Moalic (Brest); J. Roucoules, J.F. Loriferne, G. Otterbein (Bry-sur-Marne); J.F. Desson, M. Leporrier, C. Duhamel (Caen); J.M. Korach (Chalons en Champagne); B. Salles, C. Sire (Chalon/Saône); V. Herve, B. Souleau, (Clamart); J. Beytout, M. Cambon (Clermont-Ferrand); Y. Boussougant, D. Dreyfuss, X. Michon, P. Vinceneux (Colombes); G. Belkacem-Belkaki, S. Bretagne, M. Chousterman, P. Grimberg, A.S. Lascaux, A. Schaeffer, A. Sobel (Créteil); J.L. Bacri, G. Berthelot (Dieppe); A. Bonnin, M. Duong, J. Lopez, H. Portier (Dijon); M. Gauthier, O. Salmon (Evry); J. Bizet (Fresnes); J.L. Gaillard, C. Perronne (Garches); M.A. Desailly, H. Maisonneuve (La Roche-sur-Yon); J.P. Bedos, J. Doll, O. Eloy, J.C. Ghnassia, S. Roussin-Bretagne (Le Chesnay); C. Brocard, P. Guiffault, A. Layet, A. Morel (Le Havre); F. Botterel, P. Bouree, J.F. Delfraissy, Y. Kertaimont, P. Lozeron, K. Rérat, G. Saïd (Le Kremlin-Bicêtre); X. Cricks (Les Mureaux); M.L. Darde, A. Jaccard (Limoges); D. Bouhour, E. Dannaoui, X. Mallet, D. Peyramond, M.A. Piens, C. Trepo (Lyon); L. Berardi, F. Tremolieres (Mantes-la-Jolie); Y. Berland, A. Blancard, L. Collet, J. Delmont, H. Gallais, X. Gamby, A. Michel Nguyen, J. Moreau, N. Petit, J.M. Sainty, J. Sampol-Roubicek (Marseille); M. Bietrix, M. Nezri (Martigues); A. Fiacre, S. Levy (Meaux); C. Chandesris, X. La Torre (Montargis); P. Andres, E. Billaud, F. Boiffin, M. Hamidou, O. Morin, B. Planchon, P. Poirier, F. Raffi, D. Villers (Nantes); Ph. Clevenbergh, F. De Salvador, P. Dellamonica, X. Durand, M. Gari-Toussaint (Nice); A. Romaru, M. Texereau (Niort); L. Bret, T. Prazuk (Orléans); X. Bernard, Y. Pacheco (Pierre-Bénite); B. Becq-Giraudon, C. Kauffmann-Lacroix, J.C. Meurice, T. Pasdeloup (Poitiers); J. Deville, D. Toubas (Reims); C. Arvieux, F. Cartier, S. Chevrier, B. Degeilh, T. Frouget, C. Guiguen, P. Le Cavorzin, C. Michelet, V. Noyon (Rennes); P. Abboud, P. Brasseur, J. Leroy, J.F. Muir (Rouen); P. Babinet, F. Fraisse, N. Godineau, S. Hamane, P. Margent, D. Mechali, M. Thuong (Saint-Denis); C. Soler, (Saint-Mandé); B. Hery, J.Y. Leberre (Saint-Nazaire); A. Gregory, O. Prevot (Saint-Julien-en-Genevois); D. Christmann, J. Waller (Strasbourg); O. Bletry, P. Cahen, D. Zucman (Suresnes); B. Fortier, (Toul); X. Aubert, S. Chadapaud, X. Delbeck, A. Lafeuillade, X. Raoult (Toulon); E. Bonnet, S. Cassin, A. Gadroy, M.D. Linas, J.F. Magnaval, P. Massip, L. Prudhomme, L. Sailler (Toulouse); V. Baclet, C. Coignard, Y. Mouton, I. Ravaux (Tourcoing); C. Eloy, A. Fur, L. Rezzouk (Troyes); C. Fontier, E. Mazards (Valenciennes); M.F. Biava, P. Canton, L. Kures, C. Rabaud (Vandoeuvre-les-Nancy); D. Vittecocq (Villejuif); S. Dellion, O. Patey (Villeneuve-St-Georges); and in Paris : J.P. Bedos, O. Benveniste, C. Bouchard, S. Belaich, C. Carbon, C. Chochillon, J.P. Coulaud, V. Descamps, X. Duval, C. Leport, F. Lheriteau, P. Longuet, H. Mouas, F. Vachon, J.L. Vilde, P. Yeni (Hôpital Bichat-Claude Bernard); V. Lavarde, C. Piketty (Hôpital Broussais); B. Christoforov, J. Dupouy-Camet, J.P. Luton (Hôpital Cochin); N. Desplaces, G. Raguin (Hôpital de La Croix-Saint-Simon); P. Chevalier, M. Kazatchkine, V. Lavarde, A. Meyrier (Hôpital Européen Georges Pompidou); A. Bernadou, M. Cornet, J.P. Marie S. Oudart (Hôpital de l'Hôtel-Dieu); M. Gayraud, Y. Pean (Institut Mutualiste Montsouris); C. Aznar, B. Dupont, H. Poncelet, Karine Sitbon, Amaury de Gouvelho, Clarisse Loyer (Hôpital de l'Institut Pasteur); P. Berche, B. Dupont, V. Mathé, (Hôpital Necker-Enfants Malades); L. Baril, P. Bossi, F. Bricaire, J. Carrière, A. Datry, S. Herson, M. Jouan, M. Levy-Soussan, C. Mouquet, B. Orcel, M.M. Thiebaut (Hôpital Pitié-Salpétrière); J. Frottier, J.B. Guiard-Schmidt, B. Lebeau, J.L. Meynard, M.C. Meyohas, J.L. Poirot, P. Roux, X. Urban (Hôpital Saint-Antoine); F. Daniel, J. Gilquin, J.F. Timsit (Hôpital Saint-Joseph); J.C. Brouet, J.M. Decazes, F. Derouin, B. Eurin, J.R. Legall, C. Legendre, S. Neuville (Hôpital Saint-Louis); J.P. Escande (Hôpital Tarnier); G. Delzant, G. Kac, C. Trivalle (Hôpital Tenon).
